# Attonewton Force Resolution Measurements with Silicon Nanospheres at the Thermal Noise Limit in Ambient-Temperature Liquids

**DOI:** 10.1021/acs.nanolett.5c02698

**Published:** 2025-08-05

**Authors:** Aleksandr Kostarev, Mohammad K. Abdosamadi, Hiroshi Sugimoto, Minoru Fujii, Anita Jannasch, Erik Schäffer

**Affiliations:** † Center for Plant Molecular Biology (ZMBP), 9188 Eberhard Karls Universität Tübingen, 72076 Tübingen, Germany; ‡ Graduate School of Engineering, 12885 Kobe University, Kobe 657-8501, Japan

**Keywords:** Optical tweezers, attonewton resolution, silicon nanospheres, thermal noise limit, high-refractive-index nanoparticles, subfemtonewton detection

## Abstract

Precise force measurements are crucial for understanding fundamental physics or nanoscale interactions, such as those of molecular machines in biology. Optical tweezers are versatile force transducers for such measurements, enabling meticulous manipulation of small particles. However, achieving high-resolution, subfemtonewton force measurements under physiological conditions remains challenging due to thermal fluctuations and instrument noise. Here, we employed an ultrastable optical tweezers setup in an isolated environment with precise temperature control, which minimized instrumental noise and enabled prolonged, low-force measurements. We utilized water-suspended, high-refractive index silicon nanospheres for improved resolution and trapping stability. Our system achieved a force resolution of ≈60 aN, with a sensitivity of 2.7 fN Hz^–0.5^, allowing us to measure forces as low as 0.30 ± 0.06 fN. Our drag force measurements demonstrate the importance of optimized experimental conditions for low-force measurements, providing a robust framework for scientific investigations that require high-precision force characterization.

## Graphical abstract

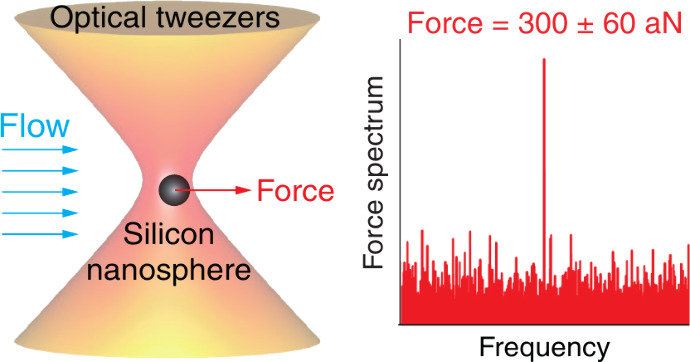

The ability to measure small forces with high precision and accuracy is essential for understanding biological and physical processes. Piconewton optical tweezers force measurements have been widely used in biophysics to study and manipulate single molecules including motor proteins, biopolymers such as DNA, and artificial micromotors.^1–5^ Some biological processes and molecules are already sensitive to subpiconewton to femtonewton forces, for example, weak molecular machines,^6^ protein polymerization,^7^ microtubule buckling,^8^ or effects that involve excluded volume or entropic forces such as DNA looping^9^ or crowding effects.^10,11^ In physics, optical tweezers have various applications from colloidal physics to probing the viscoelastic properties of complex fluids including biological matter.^12–14^ Femtonewton or even smaller forces influence phenomena such as thermophoresis,^15^ resonances in nanomechanical systems,^16^ the Casimir effect,^17,18^ or gravitational wave detection.^19^ Recent studies reported (sub)­femtonewton force measurements in aqueous solutions^9,20–34^ (Table S1). For example, Shan et al.^34^ measured forces of 0.1 ± 0.6 fN. However, their large uncertainty of ≈0.6 fN underscores the inherent difficulties in achieving precise and reliable measurements at this scale. Attonewton force resolution has only been achieved in vacuum or at cryogenic temperatures but not in biological and soft-matter systems.^16,35^ Subfemtonewton force measurements with attonewton resolution under physiological conditions still remain an outstanding challenge. Force measurements are fundamentally limited by Brownian motion. For an overdamped system, the force resolution Δ*F* (the thermal noise limit for force) defined as the smallest measurable force during the measurement time *t*_msr_ is^36^

1 $$\Delta F=\sqrt{\frac{4k_{B}T\gamma }{t_{\mathrm{msr}}}}$$

where *k*_B_ is the Boltzmann constant, *T* the absolute temperature, and γ the drag coefficient of the force probe given by Stokes drag γ = 3πη*d* = *k*_B_ *T*/*D* for a sphere with diameter *d* and diffusion coefficient *D* suspended in a medium with viscosity η far away from a surface.^37^ Under physiological conditions, the temperature and viscosity vary little. Thus, the only way to improve the force resolution is to increase the measurement time or decrease the probe size. While eq 1 is general, our focus is on force measurements with optical tweezers.

Apart from the fundamental limitation of Brownian motion, resolution is limited by instrument noise including thermal drift, optical heating, electronic noise, sound, and mechanical vibrations that affect measurements on different time scales.^4,36,38–42^ Thermal drift on long time scales and low-frequency noise eventually limit the measurement time permissible for averaging over Brownian motion and reducing fundamental noise. To overcome challenges of instrument noise and thermal drift, we developed an ultrastable optical tweezers system with temperature feedback control in an isolated environment (Figure 1, section S1 in Supporting Information, and Simmert et al.^43^ for a schematic of the used setup).^4,41–43^ The setup maintains the sample temperature with millikelvin precision for hours (Figure S1).^41,43^ With a well-equilibrated setup, surface drift is minimal with peak-to-peak sample motion of a few nanometers in all dimensions over 1000 s.^4^ Thus, our setup ensures stability for long-term measurements and minimizes instrument noise.

**Figure 1:**
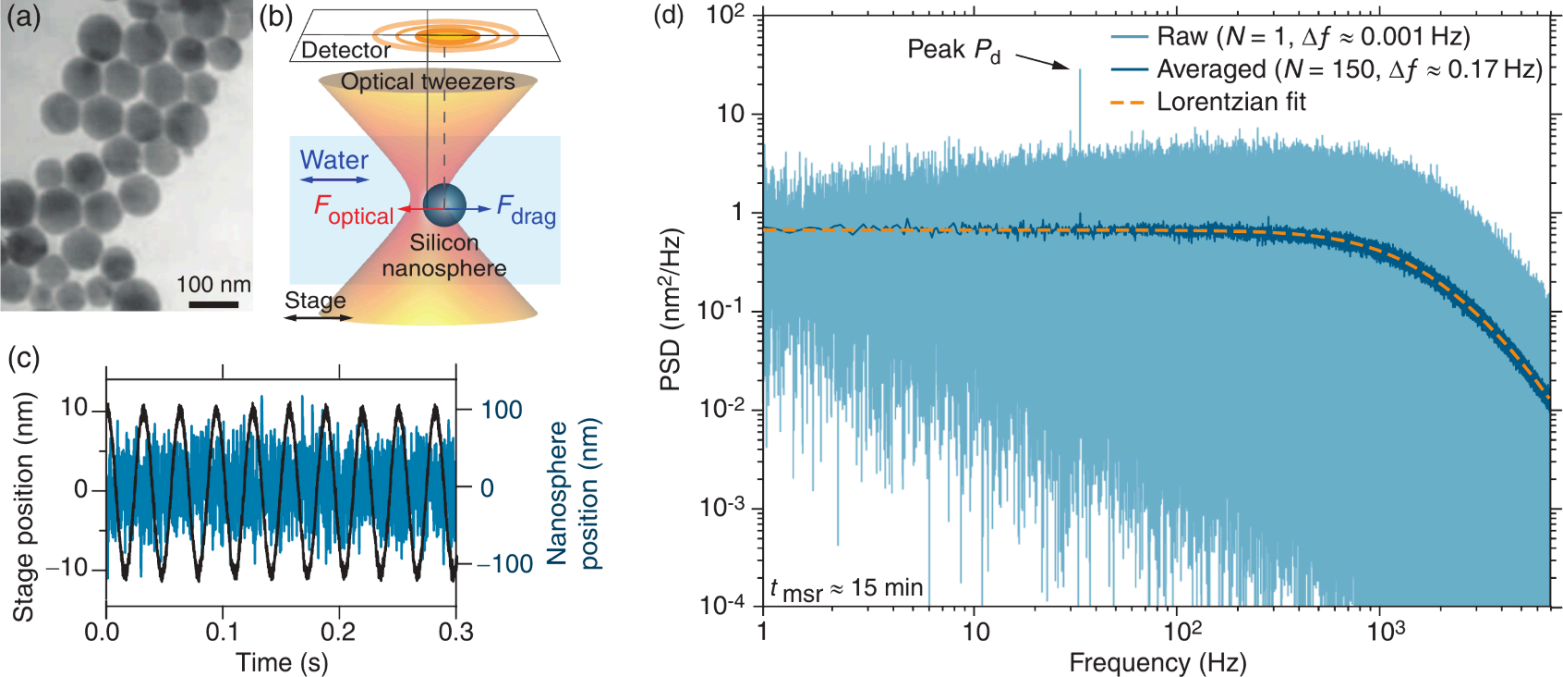
Optical tweezers force measurements with silicon nanospheres. (a) Transmission electron microscopy image of silicon nanospheres. (b) A known drag force is applied on a trapped silicon nanosphere by oscillating the aqueous sample relative to the stationary optical trap. The millikelvin precision temperature control was set to 29.500 °C. (c) Nanosphere and stage positions (blue and black line, respectively) as a function of time (*f*_*d*_ = 32 Hz). (d) Power spectral density (PSD) of the data in (c) (*t*_msr_ ≈ 15 min, light blue line with Δ*f* ≈ 0.001 Hz; dark blue line is the average of 150 PSDs of the same data broken into 150 segments with Δ*f* ≈ 0.17 Hz, and dashed dark yellow line is a Lorentzian-like fit of the hydrodynamically correct theory^37,44^ ). Note the oscillation peak at *f*_*d*_ = 32 Hz.

To minimize fundamental noise and improve the spatiotemporal and force resolution, according to eq 1, it is advantageous to reduce the probe size as much as possible. However, the trapping efficiency decreases proportional to the probe volume but increases with refractive index mismatch to the medium.^4,45^ Thus, nanosphere trapping requires a higher laser power, which can cause heating and low-frequency noise. To mitigate heating, we synthesized nanospheres with a diameter of 81 ± 26 nm (mean ± SD, *N* = 110) made of crystalline silicon, which has a complex refractive index of *n* = 3.55 + 0.00009*i* at our trapping wavelength of 1064 nm^46,47^ (Figure 1a, section S2). Thus, unlike metallic nanoparticles, which suffer from plasmonic heating and destabilizing thermal fluctuations,^48^ silicon nanospheres have a low absorption and high-refractive index that minimize optical heating and ensure stable trapping conditions with moderate laser powers.^49^ When trapping nanospheres a few micrometers away from a glass surface, we expect less than 0.5 K of heating for 100 mW of laser power in our experimental configuration.^50,51^ The high-refractive index of silicon nanospheres is also beneficial for detection, as the scattering cross-section is higher compared to silica or polystyrene spheres. Beyond their optical advantages, silicon nanospheres are chemically stable in aqueous solutions for several days.^49^ In addition, their surface chemistry allows tunable functionalization with biomolecules, chemical linkers, or coatings, enabling a wide range of applications in biophysics and nanotechnology.

To measure small forces, we trapped silicon nanospheres in deionized water, calibrated the system against thermal and drag forces,^37,44^ and asked what the lowest measurable drag force is. Employing back-focal plane interferometry,^37,52,53^ we measured the response of the trapped nanosphere to a controlled sinusoidal motion *x*(*t*) = *A* sin­(2π*f*_*d*_ *t*) of the sample chamber driven by a piezo-translation stage, where *x* is the position, *t* the time, *A* the amplitude, and *f*_*d*_ the driving frequency (Figure 1b,c and section S3). The approach is similar to the lock-in technique of Liu et al.^33^ that decouples the measurement from low-frequency instrument noise. Using a power spectral density (PSD) analysis, in which the oscillation causes a distinct peak in the one-sided PSD at the stage’s driving frequency (Figure 1d, Figure S2), we measured the displacement responsivity β^–1^ of the detector (in mV/nm), the trap stiffness κ (fN/nm), and the drag coefficient γ.^37,44^ The product α = βκ is the inverse force responsivity (in fN/mV). To rule out heating effects, we first confirmed that the drag coefficient was within error bars constant when increasing laser power consistent with our expectation of less than 0.5 K heating (Figure S3). For subsequent measurements, we used ≈80 mW in the trapping focus, selected small nanospheres, and confirmed that the measured parameters remained constant (Figure S4). Based on the measured drag coefficient, with the nanospheres relative to their size being far away from any surface,^37,44^ and the known temperature and viscosity, the diameter of the trapped nanospheres was 60 ± 3 nm with a trap stiffness of 3.1 ± 0.6 fN/nm (mean ± SD, *N* = 16, Table S2). Thus, diameters fell within the range measured by transmission electron microscopy (Figure 1a) ruling out that we trapped nanosphere clusters or larger contaminations, which are much easier to trap compared with single nanospheres. The consistency also implies that we can calculate the expected thermal noise limit based on the nanosphere diameter.

To test whether we were only limited by thermal and not instrument noise, we measured the force resolution and compared it to the expected thermal noise limit (eq 1, Figure 2, Figure S5). A precise measure for the force resolution is based on the low-frequency limit of the PSD^44^

2 $$\Delta F_{\mathrm{PSD}}=\alpha \sqrt{P_{0}\Delta f}$$

where $P_{0}=D_{V}/{\left(\pi f_{c}\right)}^{2}$ is the low-frequency plateau value of a Lorentzian (in V^2^/Hz), *D*_*V*_ the probe’s diffusion coefficient (in V^2^/s), *f*_*c*_ = κ/(2πγ) the characteristic roll-off frequency, and Δ*f* = 1/*t*_msr_ the frequency resolution. We determined the force responsivity α^–1^ and *P*_0_ by a Lorentzian-like fit of the hydrodynamically correct theory to the PSD.^37,44^ Our measurements show that the force resolution was consistent with the thermal noise limit, i.e., Δ*F*_PSD_ = Δ*F*, and decreased with increased measurement time proportional to $t_{\mathrm{msr}}^{-0.5}$ as expected from eq 1 (Figure 2, Figure S5). The proportionality constant $\alpha \sqrt{P_{0}}$ (the force sensitivity or smallest detectable force per unit bandwidth) was 2.7 fN Hz^–0.5^.

**Figure 2:**
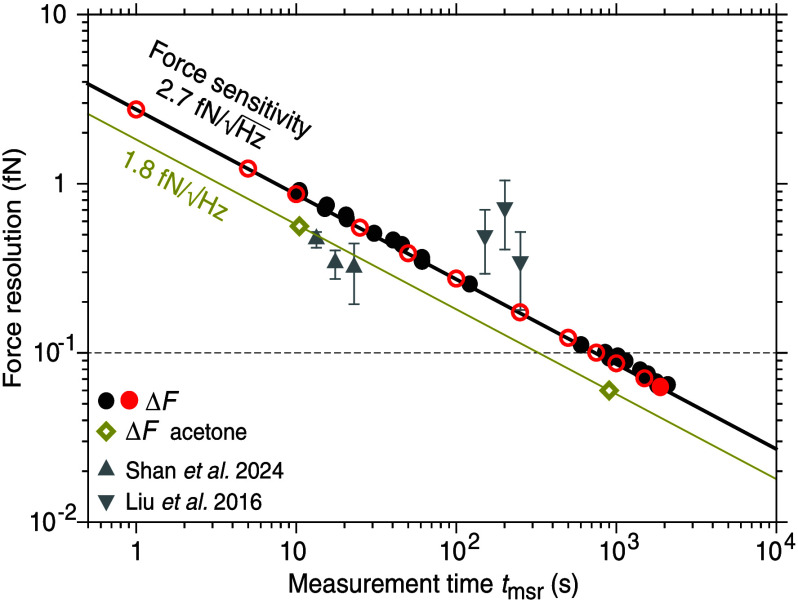
Force resolution and sensitivity. Force resolution Δ*F* as a function of measurement time *t*_msr_. Black solid circles: each data point of Δ*F*_PSD_ (eq 2) corresponds to a different nanosphere and measurement (see Table S2 for the individual measurements). Red solid circle: Δ*F*_PSD_ of the 0.3- fN measurement in Figure 3b. Red open circles: the same nanosphere with different *t*_msr_. Dark yellow open diamonds: measurements in acetone. Gray triangles: three smallest values from recent reports.^33,34^ The thermal noise limit Δ*F* (eq 1) is indicated as solid lines with their slope corresponding to the force sensitivity. The dashed horizontal line corresponds to a 100 aN resolution. Error bars are only shown when larger than the data symbols. For our measurements, relative errors were less than 3%.

For measurement times longer than ≈1000 s, the force resolution was in the attonewton range below 100 aN. For *t*_msr_ = 31 min, the force resolution was 63.0 ± 0.1 aN (red solid circle in Figure 2). For a measurement time of 10 s, we had a subfemtonewton force resolution of 0.85 ± 0.02 fN. While our *force sensitivity* was comparable to previous studies, our *force resolution* is about an order of magnitude smaller^33,34^ (compare red solid circle with gray triangles in Figure 2, Table S1). Note that it is not advantageous to average PSDs by splitting the data into segments without increasing the measurement time (section S4, Figure S6). An example is shown in Figure 1d, where the peak height at the driving frequency of the averaged PSD becomes comparable to the one of the noise peaks.

What is the lowest drag force that we can directly measure? To test the limits, accuracy, and precision of our system, we reduced the amplitude of the stage oscillation and measured the peak in the PSD and its signal-to-noise ratio (SNR) (Figure 3). With the known oscillation amplitude and drag coefficient, we know the applied drag force *F*_drag_ = γυ and can compare it to the measured one based on the oscillation-peak power in the PSD. The directly measured root-mean-square (rms) force derived from the peak PSD value *P*_*d*_ corrected for the thermal background PSD *P*_0_ is

3 $$F^{\mathrm{rms}}=\alpha \sqrt{\left(P_{d}-P_{0}\right)\Delta f}=\pi \gamma Af_{d}\sqrt{2}=F_{\mathrm{drag}}^{\mathrm{rms}}$$

where we assumed that *f*_*d*_ ≪ *f*_*c*_.^44^ For the same nanosphere with stepwise reduced oscillation amplitude, we measured femtonewton forces down to 0.9 ± 0.1 fN (*F* ^rms^ ± Δ*F* with SNR = *F* ^rms^/Δ*F* = 8, Figure 3a). Since the PSD peak power is proportional to the square of the force, even weak forces near the thermal noise limit can be reliably detected in the PSD. For the 0.9 fN peak, its SNR in the PSD was 63. With a longer measurement time, we measured a subfemtonewton force with attonewton resolution of 0.30 ± 0.06 fN (SNR = 4.7, Figure 3b). In a low-viscosity medium such as acetone, we could measure a smaller force of 0.26 ± 0.06 fN (SNR = 4.4) with the same resolution in half the measurement time due to the better force sensitivity of 1.8 fN Hz^–0.5^ (Figure 2, Figure 3c). The directly measured forces *F* ^rms^ corresponded to the applied force as expected from eq 3 $\left(F^{\mathrm{rms}}=F_{\mathrm{drag}}^{\mathrm{rms}}\right)$ showing that the measurements were not only precise but also accurate (Figure 3c).

**Figure 3:**
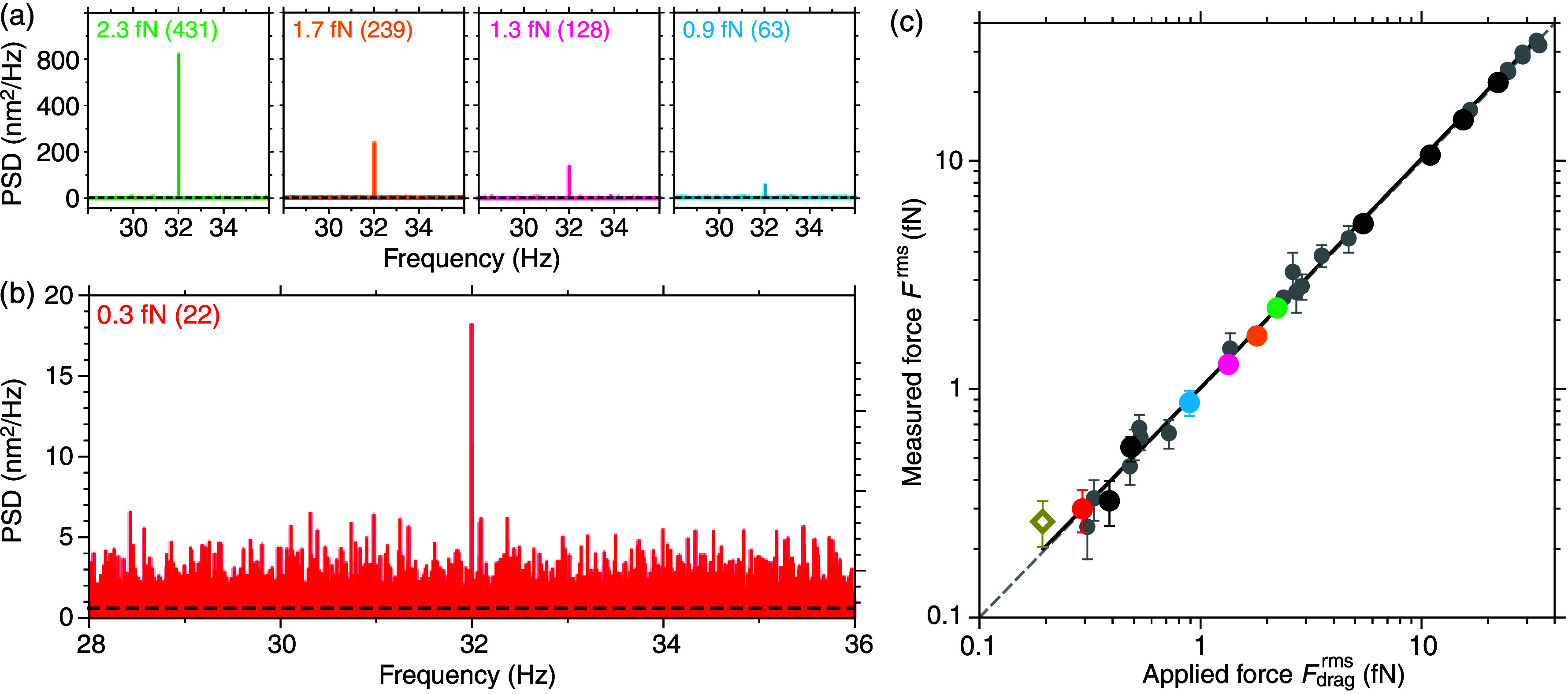
Subfemtonewton force measurements. (a) PSDs of a single nanosphere recorded with different oscillation amplitudes (*A* was 40, 32, 24, and 16 nm from left to right). Forces are indicated with peak-PSD-value SNR in parentheses (*t*_msr_ = 10 min, Δ*f* = 0.001 67 Hz, black dashed lines are Lorentzian-like fits). (b) Same nanosphere as in (a) with *A* = 4.6 nm, *t*_msr_ = 31 min, and Δ*f* = 5.3 × 10^–4^ Hz. (c) Measured force *F* ^rms^ vs the applied rms drag force $F_{\mathrm{drag}}^{\mathrm{rms}}$ (eq 3). Colored and black circles are measurements of a single nanosphere with different oscillation amplitudes (colors correspond to (a) and (b), and black circles correspond to measurements with additional oscillation amplitudes). Gray circles: each data point corresponds to a single measurement of different nanospheres trapped with various laser powers on different days. Black line: linear fit with slope 1.03 ± 0.01 and intercept 0.02 ± 0.01. Gray dashed line: $F^{\mathrm{rms}}=F_{\mathrm{drag}}^{\mathrm{rms}}.$ Dark yellow open diamond: acetone measurement (*t*_msr_ = 15 min). Error bars correspond to the force resolution shown only when larger than the data symbol (see Table S2 for individual values).

Measuring ultraweak forces under biologically relevant conditions remains challenging due to thermal fluctuations and experimental noise. The high-refractive-index silicon nanospheres trapped in ultrastable optical tweezers have an excellent force sensitivity that enables an attonewton force resolution. A further reduction in nanosphere size and SNR will either reduce the measurement time or improve the force resolution in the future. However, measurements in ambient temperature liquids are ultimately limited by eq 1. The large error of 0.6 fN in Shan et al. on a 0.1 fN force corresponds to their force resolution, highlighting the challenges of achieving precise and reliable measurements at the subfemtonewton scale with a good SNR that provides, for example, at least a 95% confidence of the measurement (SNR ≈ 2). Our 0.3 fN measurement with an SNR of 22 for the PSD peak suggests that even weaker forces could be measured under optimized conditions. Our 0.3 fN force was measured with a 3 fN/nm trap stiffness and corresponds to detecting a 1 Å rms displacement in a 220 nm peak-to-peak (6 SD) background of Brownian motion illustrating that molecular scale displacements and subfemtonewton forces can be measured under the noisy conditions in ambient liquids.

Using, for example, click chemistry^54^ or lipid bilayer coatings,^4^ we expect that silicon nanospheres can be functionalized with biomolecules, such as DNA or motor proteins. Optical trapping experiments using germanium nanospheres have shown that laser powers up to 500 mW do not cause photodamage on biomolecules.^4^ As silicon absorbs much less compared to germanium at 1064 nm, less heating is expected, which may allow even higher trapping powers. Alternatively, larger spheres up to a diameter of about 260 nm are expected to be trappable^55^ that would need less power to achieve the same trap stiffness at the price of a lower resolution. Compared with germanium, silicon nanospheres are more robust to synthesize and stable in aqueous solutions. Thus, they are promising probes for applications in biophysics and nanotechnology that require high spatiotemporal and/or force resolution. While nanosphere diameters can be reduced further, limits in trapping power of conventional optical tweezers with respect to trap stiffness and maximum forces will quickly be reached because of the cubic dependence on the probe size. For example, reducing the diameter by a factor of 2, in our case to a diameter of 30 nm, would require 8 times more laser power to achieve the same trap stiffness. Using less power weakens the trap and increases the probability that particles escape the trap due to Brownian motion. For our nanospheres, we required about 25 mW for stable trapping over minutes to hours consistent with calculations.^55^ Plasmonic tweezers are promising to push the spatiotemporal and force limits further as they can trap single proteins without the need of a nanosphere handle at comparable laser powers to the ones used here.^56,57^ However, apart from limitations on the experimental geometry for biophysical measurements, quantification of displacements and forces, while accounting for heating effects, is still challenging in plasmonic tweezers.^57^

Our advancements exceed prior benchmarks for weak force detection (Table S1), opening new possibilities for studying nanoscale interactions with unparalleled precision. This ability has implications in biophysics, nanotechnology, and fundamental physics. Our findings lay the foundation for discoveries in force spectroscopy and related fields.

## Supplementary Material



## Notes

Section S1 on setup and data acquisition; section S2 on silicon nanosphere synthesis and sample preparation; section S3 on experimental conditions and data analysis; section S4 on force resolution and averaging of PSDs; Figures S1–S6; Tables S1 and S2 (PDF)
