# Supplementary material for: Attonewton Force Resolution Measurements with Silicon Nanospheres at the Thermal Noise Limit in Ambient-Temperature Liquids
Source: Nano Lett. 2025 Aug 5;25(36):13476–81. doi: 10.1021/acs.nanolett.5c02698 (PMC12426940; doi:10.1021/acs.nanolett.5c02698)
Supplement: Supplementary file 1 [file nl5c02698_si_001.pdf]

SUPPORTING INFORMATION

Attonewton force resolution measurements with silicon nanospheres at the thermal noise limit in ambient-temperature liquids

Aleksandr Kostarev,[†,¶] Mohammad K. Abdosamadi,[†,¶] Hiroshi Sugimoto,[‡] Minoru Fujii,[‡] Anita Jannasch,[*,†] and Erik Schäffer[*,†]

†*Center for Plant Molecular Biology (ZMBP), Eberhard Karls Universität Tübingen, 72076 Tübingen, Germany*

‡*Graduate School of Engineering, Kobe University, Kobe, 657-8501, Japan*

¶*These authors contributed equally*

E-mail: anita.jannasch@uni.tuebingen.de; erik.schaeffer@uni.tuebingen.de

Table of Contents

Supporting text 2

Section S1: Setup & data acquisition 2

Section S2: Silicon nanosphere synthesis & sample preparation 2

Section S3: Experimental conditions & data analysis 3

Section S4: Force resolution & averaging of PSDs 4

Supporting figures 6

Supporting tables 10

References 12

Supporting text

S1 Setup & data acquisition

Measurements were performed in a custom-built, single-beam optical tweezers setup described in detail here.[1] Briefly, the optical tweezers are combined with interference reflection microscopy[1,2] (IRM) that provides sufficient contrast to image single nanospheres.[3] To ensure high stability, the temperature was controlled using a feedback system with millikelvin precision, set to 29.500 °C (Fig. S1).[1,4] The temperature control system enables measurements at temperatures up to 37 °C.[4] The setup is mounted on an active vibration isolation table[1] and enclosed in a custom-built chamber.[5] The nanospheres were trapped using a 1064-nm laser. The power spectral density (PSD) of the trapped nanosphere motion was computed from time traces[6] recorded from a quadrant photo diode (QP45-Q HVSD, First Sensor AG, Germany) by back-focal-plane interferometry with a sampling rate of $25600\,\mathrm{s}^{-1}$. The piezo-translation stage (LFHS3, Piezoconcept, Bron, France, updated since[1]) was oscillated at 32 Hz with various, sub-nanometer-precision amplitudes to apply a controlled drag force on the nanospheres. Stage displacements were measured and recorded from the stage's sensor output simultaneously with the photo diode signals.

S2 Silicon nanosphere synthesis & sample preparation

Silicon nanospheres were synthesized via high-temperature annealing of silicon monoxide, followed by hydrofluoric acid etching and dispersion in solvent described in detail here.[7,8] The diameter measured by transmission electron microscopy was 81 ± 26 nm (Mean ± SD, $N = 110$, inset Fig. 1a). The nanospheres were produced as powder and were dissolved in pure 99.8% absolute ethanol for storage. Before the experiment, the nanospheres were diluted in purified Type 1 water (18.2 MΩ cm, Nanopure System MilliQ reference with a Q-POD and Biopak filter) and sonicated on a table sonicator for 2 minutes. Then the diluted

sample was introduced into a custom flow cell assembled from coverslips. Coverslips were cleaned once with the detergent Mucasol and twice with anhydrous ethanol. Two Parafilm M stripes were sandwiched between the bottom (22×22 mm, #1 1/2, Corning 2850-22) and top coverslip (18×18 mm, #0, Menzel-Gläser) forming 1–2-mm wide and ≈100-µm high channel between them. The flow cell was briefly heated to 140 °C to melt the Parafilm layer and form a stable channel. After addition of the sample solution in the channel, its openings were sealed with nail polish to prevent evaporation-induced flows. Afterwards the sample flow cell was mounted in the optical tweezers setup for at least one hour prior to measurements to ensure stable thermal conditions. Since the diameter affects resolution, we aimed to trap the smallest nanospheres visible in IRM, resulting in an average diameter of 60 ± 3 nm for the analyzed, trapped nanospheres.

S3 Experimental conditions & data analysis

To minimize surface effects of the drag coefficient,[9] nanospheres were trapped and measured more than 4 µm away from the surface. To quantify low-frequency instrument noise and verify that the observed peak at 32 Hz was due to our applied drag force and not caused by laser fluctuations, electronic noise or other influences, we measured the detector signal without a trapped nanosphere (Fig. S2). Only low-frequency noise (< 1 Hz) and peaks at high frequencies (> 7 kHz) in the PSD of the nanosphere originated from instrument noise. At the stage driving frequency of 32 Hz, instrument noise was more than two orders of magnitude below the measurement signal. The drag coefficient did not depend on trapping power confirming that no significant amount of heating due to absorption of the silicon nanosphere or the surrounding water was present (Fig. S3). Increased temperatures would decrease the viscosity and drag coefficient. Measurements in Fig. 2 (red circles) and Fig. 3 (colored circles) are based on data recorded with a single nanosphere continuously trapped over 2 h with 80 mW trapping power in the laser focus. Between drag force measurements,

several calibration measurements were performed with a 615 nm oscillation amplitude, a 4 Hz frequency resolution, and averaging of 40 PSDs.[6] During the 2 h-measurement, the drag coefficient (0.45 ± 0.01 nNs/m), trap stiffness (3.1 ± 0.1 fN/nm), inverse displacement responsivity (27.1 ± 0.7 nm/mV), and inverse force responsivity (84.2 ± 0.7 fN/mV), remained constant (Fig. S4). For each individual nanosphere, the uncertainties in diameter and trap stiffness were less than 5 % based on fitting errors. These calibration controls show that the setup was stable and confirmed that no additional nanosphere or contamination was trapped (Table S2).

S4 Force resolution & averaging of PSDs

Instead of using the low-frequency limit of the PSD to calculate the force resolution (Eq. 2), an alternative approach is based on the standard deviation (SD) of the individual power-spectral values $P(f)$ at a certain frequency f. Each value $P(f)$ is exponentially distributed with its standard deviation $\sigma[P(f)]$ being equal to its mean value.[10,11] Therefore, we can also determine the force resolution based on the noise in the plateau region (assuming that $f_d \ll f_c$)

$$\Delta F_{\mathrm{SD}} = \alpha \sqrt{\sigma \left[P(f \mid f_d - \tfrac{1}{2} f_b \leq f \leq f_d + \tfrac{1}{2} f_b) \right] \Delta f}, \tag{S1}$$

where $\sigma[\ldots]$ denotes the standard deviation and f_b is the bandwidth over which the standard deviation is calculated. We used $f_b = 40$ Hz and excluded the peak at f_d. Our measurements show that the estimator for the force resolution ΔF_{PSD} was more precise compared to ΔF_{SD} but both estimates were consistent with the thermal noise limit (Fig. S5) and deviated less than 6 % from it.

Since the signal-to-noise ratio (SNR) of individual PSD values $P(f)$ is one, averaging over N power spectra reduces noise on the individual PSD values $P(f)$.[6,10,11] Averaging of PSDs implies that the total measurement time t_{msr} is divided into N segments each of duration t_{msr}/N. Thus, the frequency resolution for an individual PSD gets worse with N according

to $\Delta f = N/t_{\text{msr}}$. The force resolution estimate of ΔF_{PSD} (Eq. 2) scales with the square root of the average power $P_0 \Delta f$ of the Lorentzian plateau value in the low-frequency limit. Since we account for the small bias of the fit value P_0 on N during the fitting procedure,[11,12] its mean fit value does not depend on N. Thus, because the frequency resolution increases proportional to N, ΔF_{PSD} increases with the square-root of N, $\Delta F_{\text{PSD}} = \Delta F_1 \sqrt{N}$, where ΔF_1 is the force resolution for a non-averaged, single PSD. Since our applied drag force does not depend on whether we average PSDs, the power in the peak at our driving frequency $P_d \Delta f$ is constant and independent of N, while Δf increases linearly with N. Therefore, the height of the peak P_d decreases with the inverse of N, $P_d \propto 1/N$ that the power in the peak remains constant. Because averaging decreases the peak height, it will disappear in the noise upon averaging, which can be seen in Fig. 1d for the averaged PSD data. The SNR of the peak height will decrease accordingly.

Interestingly, our other estimate for the force resolution ΔF_{SD} (Eq. S1) scales differently with N. By averaging PSDs, the standard deviation of the individual PSD values reduces with the square root of N, $\sigma[P(f)]/\sqrt{N}$. Thus, the force resolution scales with N as

$$\Delta F_{\text{SD}} \propto \sqrt{\frac{\sigma\left[P(f)\right]}{\sqrt{N}} \frac{N}{t_{\text{msr}}}} \propto \sqrt[4]{N}. \tag{S2}$$

We determined both force resolution estimators for an increasing number of averaged PSDs (Fig. S6). Both estimators scaled and increased as expected with N. Thus, the best force resolution is achieved without averaging for a single PSD that has the best frequency resolution. Another way of thinking about it is, that the least amount of Brownian noise is contained in the smallest bandwidth of the best frequency resolution.

Supporting figures

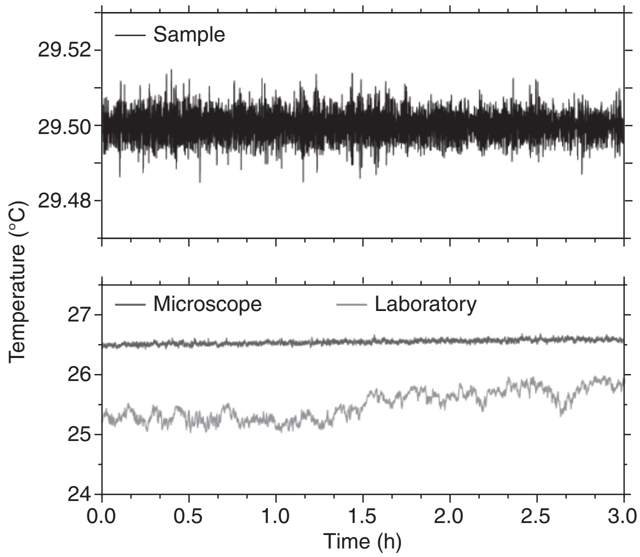

Figure S1: Temperature during the experiment of the sample (measured at the top of the oil-immersion objective,[4] top, black, $29.500 \pm 0.003\,^{\circ}\mathrm{C}$ [mean ± SD]), the custom-built microscope body inside the setup chamber (bottom, dark grey, $26.56 \pm 0.04\,^{\circ}\mathrm{C}$), and the outside laboratory room (bottom, light grey, $25.53 \pm 0.25\,^{\circ}\mathrm{C}$), indicating stable conditions throughout.

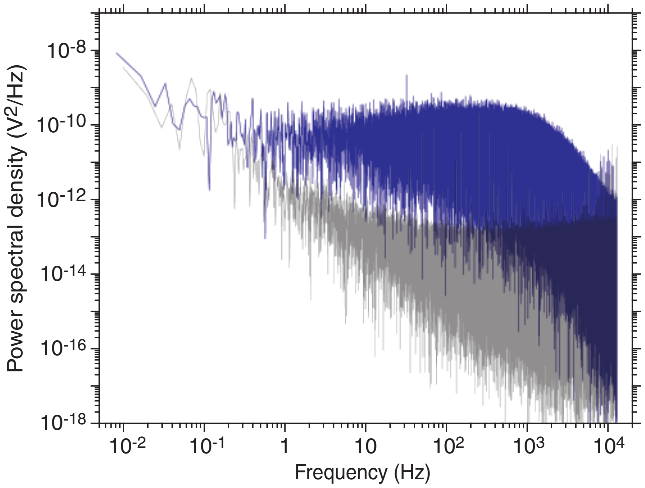

Figure S2: PSD of a trapped silicon nanosphere (blue) and the laser signal without a trapped nanosphere but with the same power (grey, half-transparent). The data from the nanosphere is the same as in Fig. 1d but not calibrated and plotted with an extended frequency range.

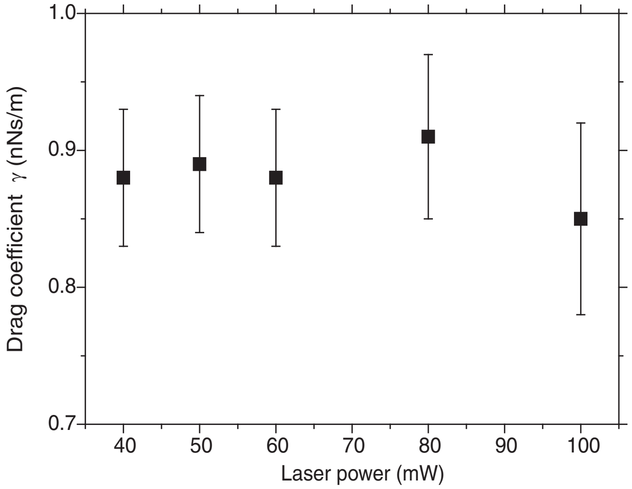

Figure S3: Measured drag coefficient γ of a single, trapped silicon nanosphere with diameter $d = 77 \pm 3\,\text{nm}$ as a function of the laser power in the trapping focus. For each calibration measurement, we averaged 40 PSDs with $\Delta f = 8\,\text{Hz}$, $A = 615\,\text{nm}$, and $f_d = 32\,\text{Hz}$.

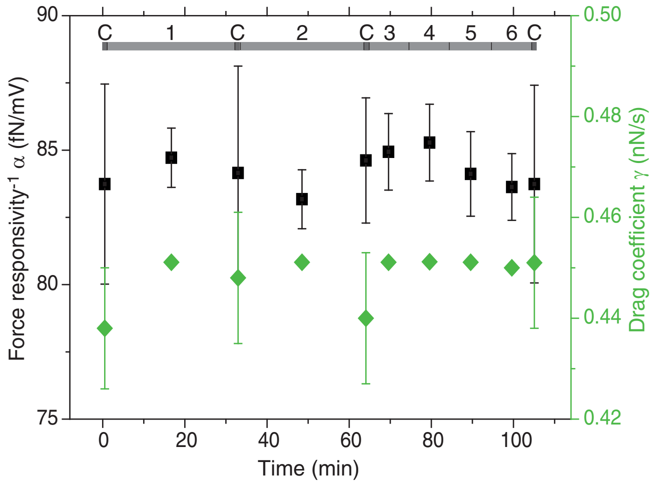

Figure S4: Inverse force responsivity α (black squares, left vertical axis) and drag coefficient γ (green diamonds, right vertical axis) for single nanosphere measurements shown in Fig. 3a,b. The nanosphere remained trapped for 2 h during repeated measurements. The gray bar at the top with dark gray markings indicates the measurement duration (t_{msr}) for each data point (measurement 1–6). Between drag force measurements, several calibration (C) measurements were performed.

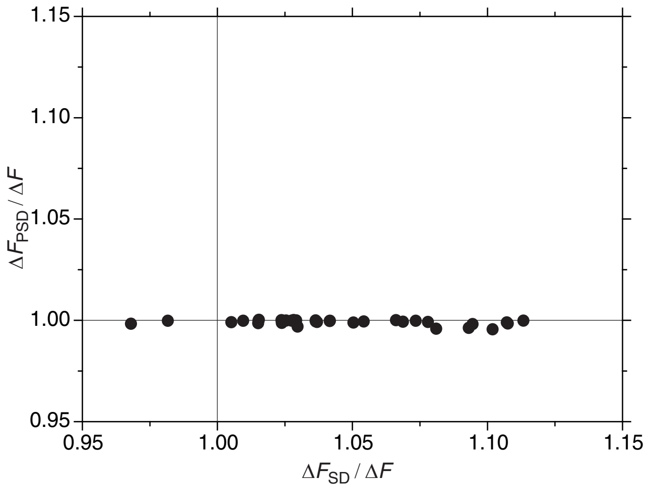

Figure S5: Force resolution ΔF_{PSD} (Eq. 2) plotted versus ΔF_{SD} (Eq. S1) both normalized by the thermal noise limit ΔF (Eq. 1). ΔF_{PSD} was more precise than ΔF_{SD} but both estimates were consistent with the thermal noise limit. Data are from 16 different nanospheres with different measurement times.

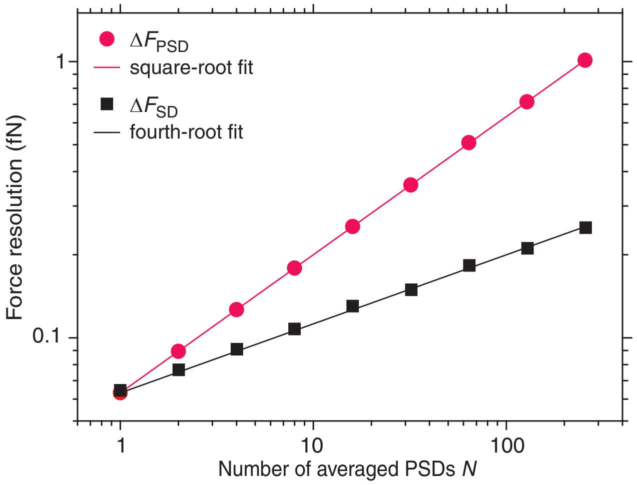

Figure S6: Force resolution ΔF_{PSD} (red circles) and force resolution ΔF_{SD} (black squares) as a function of the number of averaged PSDs N fitted to power laws (see Sect. S4 for details). Data corresponds to the 0.3-fN measurement shown in Fig. 3b. The red and black line are fits of $\Delta F = \Delta F_1 N^p$ with the exponent p equal to $\frac{1}{2}$ and $\frac{1}{4}$, respectively. Both fits resulted in the same force resolution for a single PSD of $\Delta F_1 = 63\,\text{aN}$, corresponding to the thermal noise limit and force resolution of the non-averaged PSD.

Supporting tables

Table S1: Sub-piconewton force measurements. Displayed are the smallest measured force (F), force resolution (ΔF), force sensitivity ($S_F^{1/2}$), and type of the applied force (origin of the force) for exemplary publications.

F (fN)	ΔF (fN)	$S_F^{1/2}$ (fN/Hz$^{0.5}$)	Origin of the force	Reference
240		≈24	Optical pressure	Pollard 2010[13]
200			Optical pressure	Fontes 2005[14]
≈100			Optical pressure	Hohng 2007[15]
25.6	2.2 fN		Optical pressure	Rohrbach 2005[16]
25			Dielectrophoresis	Imasato 2008[17]
20			Thermophoretic motion	Helden 2015[18]
	10		Thermal motion	Gibson 2008[19]
45	10		Depletion force	Rudhardt 1999[20]
8-12			Magnetic force	Stoev 2021[21]
10			Depletion force	Prieve 1999[22]
	≈8		Optical pressure	Marago 2008[23]
≈8	≈1.8		Thermally activated state transition	Chen 2012[24]
	≈5		Surface electrical charges	Sainis 2008[25]
	2.2		Optical pressure	Zensen 2016[26]
10	<1 fN		Optical pressure	Liu 2016[27]
0.1	0.6	1.6	Electrophoresis forces	Shan 2024[28]
**0.3**	**0.06**	**2.7**	**Drag force**	**Our measurement**

Table S2: Parameter values of the data plotted in Fig. 2 and Fig. 3. Listed are the measured force (F^{rms}), applied drag force (F_{drag}), force resolution (ΔF_{PSD} and ΔF_{SD}), signal-to-noise ratio for the PSD peak ($\mathrm{SNR}_{\mathrm{PSD}}$) and for force ($\mathrm{SNR}_F$), fitted zero-frequency limit of Lorentzian plateau value (P_0), amplitude of oscillation (A), frequency resolution (Δf), trap stiffness (κ), detector displacement responsivity (β^{-1}, different neutral density filters in front of the QPD detector led to different magnitudes of values), measured nanosphere diameter (d), and measurement time (t_{msr}). The last two rows correspond to the measurements in acetone. The maximum coefficient of variation for κ and β did not exceed 1%.

F^{rms} (fN)	$F^{\mathrm{rms}}_{\mathrm{drag}}$ (fN)	ΔF_{PSD} (fN)	ΔF_{SD} (fN)	$\mathrm{SNR}_{\mathrm{PSD}}$	SNR_F	P_0 (nm^2/Hz)	A (nm)	Δf (Hz)	κ (fN/nm)	β (nm/mV)	d (nm)	t_{msr} (s)
0.30	0.29	0.063	0.065	22.4	4.7	0.76	4.6	0.00053	3.15	26.9	59.3	1886.8
0.32	0.39	0.073	0.076	19.9	4.5	0.74	5.7	0.00067	3.28	25.1	62.7	1500.8
0.56	0.49	0.065	0.066	75.0	8.7	0.76	7.6	0.00055	3.15	26.4	59.3	1808.0
0.88	0.89	0.112	0.116	62.6	7.9	0.80	13.9	0.00167	3.06	27.7	59.3	600.3
1.26	1.34	0.112	0.117	127.8	11.3	0.76	20.9	0.00167	3.11	27.2	59.3	600.4
1.72	1.80	0.112	0.116	238.6	15.4	0.74	28.1	0.00166	3.19	26.4	59.3	600.8
2.32	2.22	0.112	0.113	430.6	20.8	0.73	34.6	0.00167	3.20	26.1	59.3	600.5
0.23	0.31	0.065	0.068	12.8	3.6	0.87	4.1	0.00048	3.21	115.3	70.3	2100.7
0.33	0.33	0.068	0.070	24.2	4.9	0.30	4.8	0.00056	5.26	72.1	64.3	1777.8
0.65	0.72	0.094	0.096	47.4	6.9	0.53	10.7	0.00111	3.87	98.1	62.3	900.9
0.58	0.50	0.090	0.100	41.7	6.5	0.37	6.4	0.00088	5.00	69.4	72.4	1139.5
0.68	0.53	0.096	0.097	50.0	7.1	0.71	6.6	0.00098	3.64	101.2	74.4	1020.8
0.62	0.54	0.075	0.078	67.1	8.2	0.71	7.1	0.00064	3.55	98.8	70.3	1571.6
0.46	0.48	0.080	0.085	33.6	5.8	1.59	6.3	0.00071	2.37	113.2	70.3	1410.0
2.55	2.37	0.101	0.104	637.0	25.2	0.97	32.1	0.00118	2.99	106.0	68.3	850.5
3.91	3.53	0.438	0.445	79.5	8.9	2.13	47.8	0.02210	2.02	163.1	68.3	45.2
2.87	2.87	0.368	0.383	60.7	7.8	2.58	41.3	0.01654	1.78	180.3	64.3	60.5
15.48	15.38	0.751	0.824	425.1	20.6	1.81	208.1	0.06536	2.18	166.0	68.3	15.3
30.58	28.41	0.914	1.012	1118.6	33.4	1.78	384.3	0.09709	2.20	165.9	68.3	10.3
1.52	1.36	0.256	0.275	35.2	5.9	0.80	20.2	0.00831	3.15	115.8	62.3	120.4
2.70	2.71	0.511	0.546	28.0	5.3	0.76	40.2	0.03295	3.22	115.6	62.3	30.3
4.65	4.67	0.622	0.690	55.8	7.5	0.74	69.3	0.04902	3.26	115.2	62.3	20.4
25.20	24.51	0.880	0.955	820.1	28.6	1.69	363.6	0.09851	2.16	164.8	62.3	10.2
29.46	28.49	0.655	0.707	2020.0	44.9	0.55	385.4	0.04951	3.99	77.9	68.3	20.2
25.67	24.36	0.885	0.859	840.4	29.0	0.95	350.1	0.09616	2.92	120.0	64.3	10.4
34.50	32.95	0.466	0.478	5477.9	74.0	0.95	445.8	0.02506	3.02	85.4	68.3	39.9
32.99	33.90	0.895	0.924	1358.8	36.9	0.67	487.2	0.09852	3.47	108.7	64.3	10.2
3.31	2.61	0.716	0.785	21.4	4.6	1.03	40.1	0.06712	2.73	131.7	60.3	14.9
5.40	5.43	0.349	0.355	239.2	15.5	0.76	86.2	0.01650	3.11	26.7	58.3	60.6
10.82	10.91	0.350	0.363	956.1	30.9	0.76	173.1	0.01654	3.12	26.6	58.3	60.4
17.09	16.49	0.350	0.352	2387.0	48.9	0.77	261.4	0.01653	3.09	26.7	58.3	60.5
22.62	22.09	0.350	0.368	4179.2	64.6	0.77	350.3	0.01656	3.10	26.6	58.3	60.4
0.26	0.19	0.060	0.064	19.2	4.4	1.19	7.0	0.00111	1.66	313.1	69.4	900.6
12.72	12.89	0.564	0.625	509.4	22.6	0.90	462.4	0.09708	1.90	270.7	69.4	10.3

References

(1) Simmert, S.; Abdosamadi, M. K.; Hermsdorf, G.; Schäffer, E. LED-based interference-reflection microscopy combined with optical tweezers for quantitative three-dimensional microtubule imaging. *Opt. Express* **2018**, *26*, 14499–14513.

(2) Mahamdeh, M.; Simmert, S.; Luchniak, A.; Schäffer, E.; Howard, J. Label-free high-speed wide-field imaging of single microtubules using interference reflection microscopy. *J. Micros.* **2018**, *272*, 60–66.

(3) Sudhakar, S.; Abdosamadi, M. K.; Jachowski, T. J.; Bugiel, M.; Jannasch, A.; Schäffer, E. Germanium nanospheres for ultraresolution picotensiometry of kinesin motors. *Science* **2021**, *371*, eabd9944.

(4) Mahamdeh, M.; Schäffer, E. Optical tweezers with millikelvin precision of temperature-controlled objectives and base-pair resolution. *Opt. Express* **2009**, *17*, 17190–17199.

(5) Hermsdorf, G. L.; Szilagyi, S. A.; Rösch, S.; Schäffer, E. High performance passive vibration isolation system for optical tables using six-degree-of-freedom viscous damping combined with steel springs. *Rev. Sci. Instrum.* **2019**, *90*, 015113.

(6) Tolić-Nørrelykke, S. F.; Schäffer, E.; Howard, J.; Pavone, F. S.; Jülicher, F.; Flyvbjerg, H. Calibration of optical tweezers with positional detection in the back focal plane. *Rev. Sci. Instrum.* **2006**, *77*, 103101.

(7) Hinamoto, T.; Hotta, S.; Sugimoto, H.; Fujii, M. Colloidal solutions of silicon nanospheres toward all-dielectric optical metafluids. *Nano Lett* **2020**, *20*, 7737–7743.

(8) Sugimoto, H.; Okazaki, T.; Fujii, M. Mie resonator color inks of monodispersed and perfectly spherical crystalline dilicon nanoparticles. *Adv. Optical Mater.* **2020**, *8*.

(9) Schäffer, E.; Nørrelykke, S. F.; Howard, J. Surface forces and drag coefficients of mi-

crospheres near a plane surface measured with optical tweezers. *Langmuir* **2007**, *23*, 3654–3665.

(10) Berg-Sørensen, K.; Flyvbjerg, H. Power spectrum analysis for optical tweezers. *Rev. Sci. Instrum.* **2004**, *75*, 594–612.

(11) Nørrelykke, S. F.; Flyvbjerg, H. Power spectrum analysis with least-squares fitting: Amplitude bias and its elimination, with application to optical tweezers and atomic force microscope cantilevers. *Rev. Sci. Instrum.* **2010**, *81*, 075103.

(12) Simmert, S.; Jachowski, T. A Python package to calibrate optical tweezers and analyze time-dependent signals. figshare (2020), `https://github.com/cellular-nanoscience/pyotic`.

(13) Pollard, M. R.; Botchway, S. W.; Chichkov, B.; Freeman, E.; Halsall, R. N. J.; Jenkins, D. W. K.; Loader, I.; Ovsianikov, A.; Parker, A. W.; Stevens, R.; Turchetta, R.; Ward, A. D.; Towrie, M. Optically trapped probes with nanometer-scale tips for femto-Newton force measurement. *New J. Phys.* **2010**, *12*, 113056.

(14) Adriana, F.; Selma, G.; Archimedes, J., B. de Castro; Vivaldo, M. N.; Liliana de, Y. P.; Gustavo, P. M.; Luiz, C. B.; Carlos, L. C. Determination of femto Newton forces and fluid viscosity using optical tweezers: application to Leishmania amazonensis. *Proc. SPIE* **2005**, *5699*, 419–425.

(15) Hohng, S.; Zhou, R.; Nahas, M. K.; Yu, J.; Schulten, K.; Lilley, D. M. J.; Ha, T. Fluorescence-force spectroscopy maps two-dimensional reaction landscape of the Holliday junction. *Science* **2007**, *318*, 279–283.

(16) Rohrbach, A. Switching and measuring a force of 25 femtoNewtons with an optical trap. *Opt. Express* **2005**, *13*, 9695–9701.

(17) Imasato, H.; Yamakawa, T. Measurement of dielectrophoretic force by employing controllable gravitational force. *J. Electrophor.* **2008**, *52*, 1–8.

(18) Helden, L.; Eichhorn, R.; Bechinger, C. Direct measurement of thermophoretic forces. *Soft Matter* **2015**, *11*, 2379–2386.

(19) Gibson, G. M.; Leach, J.; Keen, S.; Wright, A. J.; Padgett, M. J. Measuring the accuracy of particle position and force in optical tweezers using high-speed video microscopy. *Opt. Express* **2008**, *16*, 14561–14570.

(20) Rudhardt, D.; Bechinger, C.; Leiderer, P. Repulsive depletion interactions in colloid-polymer mixtures. *J. Phys.: Condens. Matter* **1999**, *11*, 10073.

(21) Stoev, I. D.; Seelbinder, B.; Erben, E.; Maghelli, N.; Kreysing, M. Highly sensitive force measurements in an optically generated, harmonic hydrodynamic trap. *eLight* **2021**, *1*, 2662–8643.

(22) Prieve, D. C. Measurement of colloidal forces with TIRM. *Adv. Colloid Interface Sci.* **1999**, *82*, 93 – 125.

(23) Maragò, O. M.; Jones, P. H.; Bonaccorso, F.; Scardaci, V.; Gucciardi, P. G.; Rozhin, A. G.; Ferrari, A. C. Femtonewton force sensing with optically trapped nanotubes. *Nano Lett.* **2008**, *8*, 3211–3216.

(24) Chen, F.-J.; Wong, J.-S.; Hsu, K. Y.; Hsu, L. Thermally activated state transition technique for femto-Newton-level force measurement. *Opt. Lett.* **2012**, *37*, 1469–1471.

(25) Sainis, S. K.; Germain, V.; Mejean, C. O.; Dufresne, E. R. Electrostatic interactions of colloidal particles in nonpolar solvents: role of surface chemistry and charge control agents. *Langmuir* **2008**, *24*, 1879–3088.

(26) Zensen, C.; Villadsen, N.; Winterer, F.; Keiding, S. R.; Lohmüller, T. Pushing nanoparticles with light — A femtonewton resolved measurement of optical scattering forces. *APL Photonics* **2016**, *1*, 026102.

(27) Liu, L.; Kheifets, S.; Ginis, V.; Capasso, F. Subfemtonewton force spectroscopy at the thermal limit in liquids. *Phys. Rev. Lett.* **2016**, *116*, 228001.

(28) Shan, X.; Ding, L.; Wang, D.; Wen, S.; Shi, J.; Chen, C.; et all., Sub-femtonewton force sensing in solution by super-resolved photonic force microscopy. *Nat. Photonics* **2024**, *8*, 3211–3216.
